# Beneficial Effects of Ants and Spiders on the Reproductive Value of *Eriotheca gracilipes* (Malvaceae) in a Tropical Savanna

**DOI:** 10.1371/journal.pone.0131843

**Published:** 2015-07-13

**Authors:** Vanessa Stefani, Tayna Lopes Pires, Helena Maura Torezan-Silingardi, Kleber Del-Claro

**Affiliations:** Programa de Pós-Graduação em Ecologia e Conservação de Recursos Naturais, Laboratório de Ecologia Comportamental e de Interações (LECI), Instituto de Biologia, Universidade Federal de Uberlândia, Uberlândia, MG, Brazil; Portland State University, UNITED STATES

## Abstract

Predators affect plant fitness when they forage on them and reduce the action of herbivores. Our study evaluates the complementary effects of spiders and ants that visit the extrafloral nectaries of *Eriotheca gracilipes *(Malvaceae) on the production of fruits and viable seeds of these savanna trees. Four experimental groups were established: control group – with free access of spiders and ants; exclusion group – spiders and ants excluded; ant group – absence of spiders; and spider group – absence of ants. The presence of ants reduced the spider richness; however, the presence of spiders did not affect the ant richness. A significantly higher number of fruits per buds were found in the presence of spiders alone or spiders and ants together (control group) compared with the absence of both predators (exclusion group). The number of seeds per fruits and seed viability were higher in the control group. This is the first study showing that spiders and ants may exert a positive and complementary effect on the reproductive value of an extrafloral nectaried plant. Mostly the impact of ants and/or spiders on herbivores is considered, whereas our study reinforces the importance of evaluating the effect of multiple predators simultaneously, exploring how the interactions among predators with distinct skills may affect the herbivores and the plants on which they forage.

## Introduction

In terrestrial communities, multitrophic interactions comprise a minimum of three trophic levels that interact among each other: plants, herbivores and their natural enemies [1, 2]. The top-down forces exerted by invertebrate predators on herbivores and their cascading effect on plants are very important for the structuring of terrestrial communities [3, 4, 5]. Among major invertebrate predators, ants exert a strong impact on the density and spatial distribution of leaf and floral herbivores, which is reflected in the reproductive capacity of the plants [6, 7, 8]. This important effect has only recently also been attributed to spiders [9, 10, 11].

Studies of ant-plant-herbivore interactions have reported that the host plant may benefit from these relationships, depending on the behavior of the ant species associated with it and on the intensity and quality of the herbivore attack [12, 13]. For example, the action of ants against the main floral herbivore of the Brazilian savanna plant *Qualea multiflora*, the beetle *Macrodactylus pumilio* Burm. (Scarabaeidae), led to a 40% increase in this plant’s fruit production [14]. Nascimento and Del-Claro [15] performed experimental manipulations under natural conditions and observed that the presence of ants that visit the extrafloral nectaries of *Chamaecrista debilis* (Fabaceae) increased its fruit production by more than 50% in relation to plants from which ants were excluded. The constant presence of ants in the vegetation is due mainly to the existence of different renewable sources of liquid food in the foliage, such as extrafloral nectaries [16], which effectively nourish the ants and allow for better ant colony development [17]. Therefore, ants, herbivores and plants establish interaction networks that influence the structure of communities in tropical environments [18, 19]. These networks may be sustained over time as a function of the sequential flowering and budding of the host plants [20]. The phenological synchronization between ants, herbivores and their host plants may determines the quantity and quality of food resources, including extrafloral nectar, directly impacting populations and communities [18, 20].

Studies of trophic interactions involving spiders and their impacts on the vegetation have increased considerably in the last few years. This is expected, considering that spiders are present in almost all terrestrial environments and occur in higher abundance in vegetation-rich areas [21]. Regarded as excellent predators, spiders also use plants as foraging substrates, exploring differences in the plant architecture and in prey capture strategies, and may be categorized into different guilds [22, 23]. Furthermore, spiders commonly prey on insect herbivores, which can result in a great decrease in herbivory rates, benefiting the host plants [11, 24]. Ruhren and Handel [24] found that the presence of Salticidae spiders on *Chamaecrista nictitans* (Caesalpiniaceae) resulted in a higher number of fruits and seeds produced in comparison to plants without spiders. In contrast, the spider *Peucetia viridans* (Oxyopidae) preys on pollinators and seed herbivores of *Haplopappus venetus* (Asteraceae), generating ambiguous effects for the host plant. The negative effect was the low number of fertilized eggs reducing the number of seeds produced, the positive effect was the low number of seeds injured by herbivores [25]. Nahas and co-workers [11] showed that, for a multitrophic system that involves the presence of ants and spiders simultaneously, these predators may have a significant complementary effect on the reduction of leaf area loss and the reduction of herbivore presence on the plants.

Tropical savannas, such as the Brazilian Cerrado, are rich in examples of plants visited simultaneously by spiders and ants; however, several aspects of these interactions are still poorly studied. The Cerrado is an ecosystem rich in plants with extrafloral nectaries and is the second largest Brazilian biome, although it is highly endangered by agricultural expansion and urban growth in the interior of the country [26]. Plants with extrafloral nectaries or with herbivorous trophobionts in the Cerrado are commonly visited by ants that effectively reduce their leaf area loss [27, 28]. More recently, it has been shown that spiders of several families (e.g., Anyphaenidae, Salticidae, Araneidae, Theridiidae, Thomisidae, and Oxyopidae) are common in Cerrado plants [29] and may visit extrafloral nectaries and attack herbivores [11]. Understanding the interactions that structure natural communities in the Cerrado is important because the simultaneous interaction of several species provides a connection among the studies of the natural history of these species, allowing studies of the community and of ecosystem patterns and processes [30, 31, 32].

The hypothesis of the present study was that ants and spiders increase the reproductive value of plants with extrafloral nectaries in the Cerrado and that they may have an isolated or a complementary effect. *Eriotheca gracilipes* (Malvaceae) trees, a species with extrafloral nectaries on its leaf blades that is abundant in Cerrado areas, was used as a model. To test our hypothesis, we addressed four main questions: (a) Is the species richness of spiders or ants affected by the co-occurrence of these predators on host plants? (b) What are the main guilds of spiders and ants species that occur on these extrafloral nectaried plants? (c) Do ants and/or spiders exert a positive impact on plant reproduction? (d) Are the outcomes of these predator-plant relationships dependent on the co-occurrence of ants and spiders in the same host plants, or it does not matter?

## Materials and Methods

### Study site

Field observations and experiments were performed from April to September 2012 in a Private Reserve of Natural Heritage (*Reserva Particular do Patrimônio Natural*–RPPN) (18°58’00” S and 48°17’30” W) belonging to the Itororó Hunting and Fishing Club of Uberlândia, located in the municipality of Uberlândia, in the state of Minas Gerais, Brazil. The Biology Institute of Universidade Federal de Uberlândia has a memorandum of understanding with CCPIU (Itororó Hunting and Fishing Club of Uberlândia), an agreement between Mr. Nilson Dias, head of CCPIU, and Dr. Kleber Del Claro, director of Biology Institute that enables ecological studies in the area. The field studies did not involve endangered or protected species. The climate of the region presents a dry season from May to September and a rainy season from October to April. The mean annual temperature is 22°C, and the total rainfall is 1500 mm per year. The predominant vegetation in the area is cerrado *sensu stricto* (see [33] for a better characterization of the area).

### Host plant

*Eriotheca gracilipes* (Malvaceae) is a tree species that may reach up to 15 m in height in Cerrado areas and occurs more commonly in Cerrado areas in the southeast of Brazil [34, 35]. This species flowers from May to August; the flowers are receptive for two or three days and are self-incompatible and thus dependent on large bees (Anthophoridae) for pollination [36]. This species possesses extrafloral nectaries at the base of the petiole, in the veins at the abaxial region of the blade of young leaves [37] and at the base of the corolla of the flowers, extending their activity until the formation of the fruit.

### Experimental manipulation

In April 2012, 60 *E*. *gracilipes* individuals were selected at a similar phenological state, at a height of between 1 and 2.5 meters, and were distant at least five meters from one another. These individuals were randomly divided into four groups of 15 individuals. After the random draw that separated the individuals, each group received a name and treatment. The control group was not manipulated. The remaining groups were subjected to the following experimental manipulations: exclusion group–removal of both spiders and ants from the plants; ant group–removal of spiders from the plants; and spider group–removal of ants from the plants.

Ants were excluded (exclusion group and spider group) manually, and after the removal, we covered the trunks with a 5 cm large adhesive paper strap and applied a layer of sticky resin over it (Tanglefoot) on the trunk of the plant 30 cm from the soil. This resin acts as a physical barrier preventing ant access to the plants. The branches of plants in the vicinity and any other structures from the environment that could act as a passage for ants were cut.

**Fig 1:**
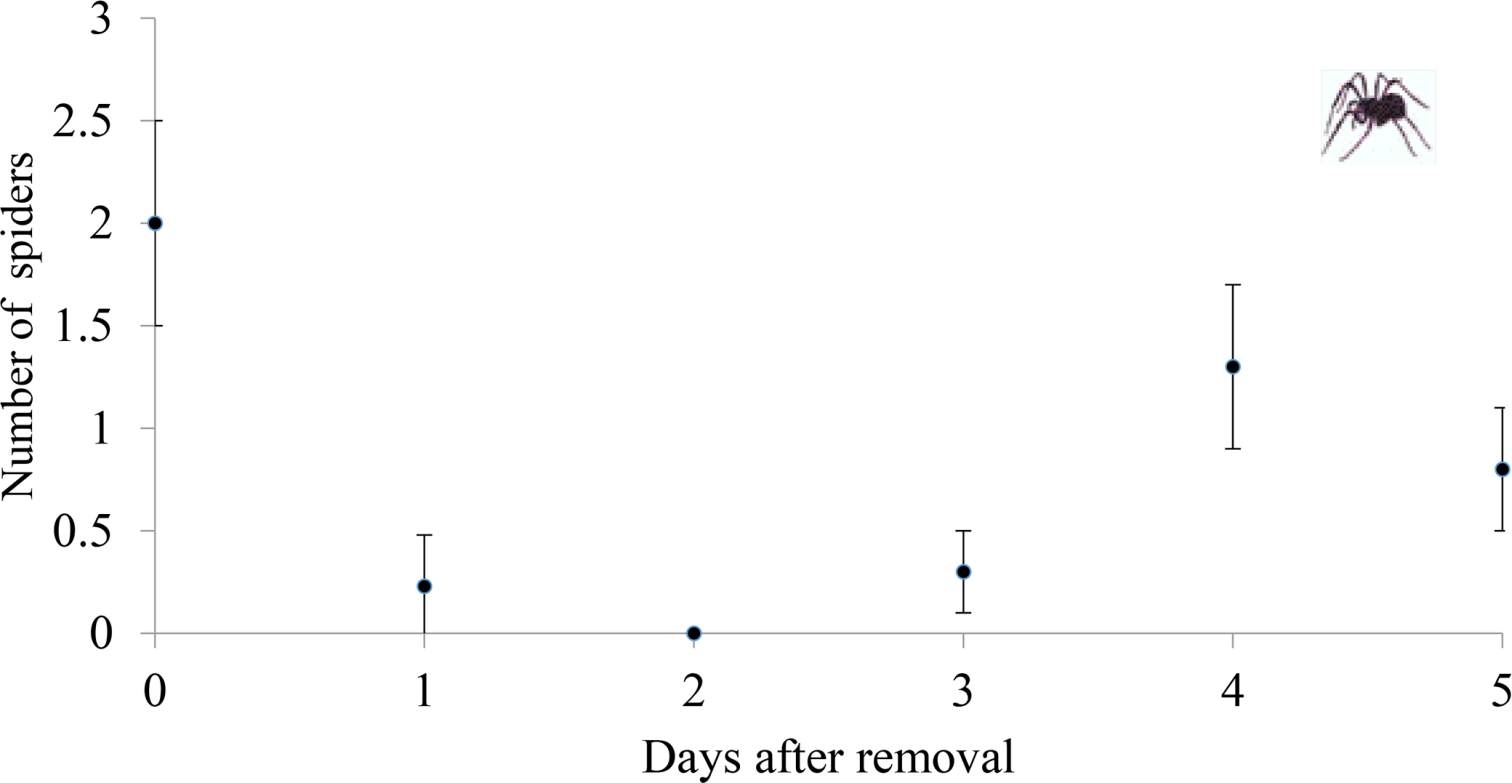
Spiders recolonization. Recolonization by spiders (mean ± standard error) removed from *Eriotheca gracilipes* plants (N = 5) over a period of five days in a removal experiment.

Spiders were excluded (exclusion group and ant group) by manual removal of all individuals every three days. This time interval was established during the pre-experimental phase (before the reproductive period of the plants), when spiders were removed from five host plants and their reestablishment was inspected for a period of five days, following the method of [11]. The recolonization during the experiment was low, and the mean number of spiders in the plants was lower during the following five days than on the first day of the experiment (Fig 1).

The individuals trees were inspected (12 days per month, once each three days), marked and monitored from the appearance of floral buds (May) until the formation of fruit (September). The number of seeds per fruit was counted, and the viability of all seeds in each experimental group was evaluated. The spider guilds, ant species, flower bud herbivores, flower herbivores and fruit herbivores were also recorded for each host plant. The spiders were divided into four guilds, according to [38, 39]: trappers (e.g., Araneidae, Theridiidae), jumpers (e.g., Salticidae and Oxyopidae), ambushers (e.g., Ctenidae, Thomisidae), and pursuers (e.g., Anyphaenidae).

### Seed viability test

We checked whether the absence and/or presence of spiders and ants influence the quantity and quality of seeds, performing a viability test. As the seeds of *E*. *gracilipes* are considered recalcitrant was used the tetrazolium test. This test is based on the alteration of enzyme activities and has been shown to be a good choice because of the speed in the determination of the viability and vigor of the seeds [40, 41, 42]. The tetrazolium test distinguishes between viable and dead tissues of the embryo on the basis of their relative respiration rate in the hydrated state [40]. In short, dehydrogenase enzymes react with substrates and release hydrogen ions to the oxidized, colorless, tetrazolium salt solution, which is changed into the red. This makes it possible to distinguish live parts, colored red, those dead ones [40, 41, 42].

When the *E*. *gracilipes* fruits began to crack, they were collected, counted and left to dry for 24 hours until they were completely open. The fruits were tagged according to manipulation of the host plant and the seeds were then counted and later immersed for staining in a solution of approximately 0.5% 2,3,5-triphenyltetrazolium chloride for 24 hours in the dark at 35°C. After this period, the seeds were analyzed individually by opening them longitudinally with a surgical blade, observing the external and internal faces between the cotyledons, and verifying the appearance of light carmine red coloration (indicating the viability of the seed) or white coloration (indicating the inviability of the seed) (see [40, 41, 42]).

### Statistical analyses

The assumptions of the parametric test were tested using Levene’s test for equality of variances and Lilliefors’ test for normality. In those cases where data did not satisfy the assumptions of a normal distribution (p < 0.05) and transformations were unable to achieve data normality, non-parametric statistical tests were used. Student’s t-test was used to compare the ant richness in the presence and absence of spiders, as well as the spider richness in the presence and absence of ants. To determine the effect of ants and spiders (together or separately) on plant productivity (numbers of fruits formed per floral buds produced; seeds per fruits; and viable seeds) in each experimental group was used the Kruskal-Wallis test with a post-hoc Dunn (Q) test.

The abundance of the families of herbivores present and their proportions during the different stages of the reproductive development of *E*. *gracilipes* (flower buds, flowers and fruits) were quantified in each experimental group. To know whether presence or absence of ants and spiders (together or separately) affected herbivores abundance during the plant reproductive period, the number of herbivores in each experimental group was quantified in buds, flowers and fruits, since the formation of first buds until the time of seeds viability tests. We also used Kruskal-Wallis test with post-hoc Dunn (Q) test to compare the groups.

## Results

**Fig 2:**
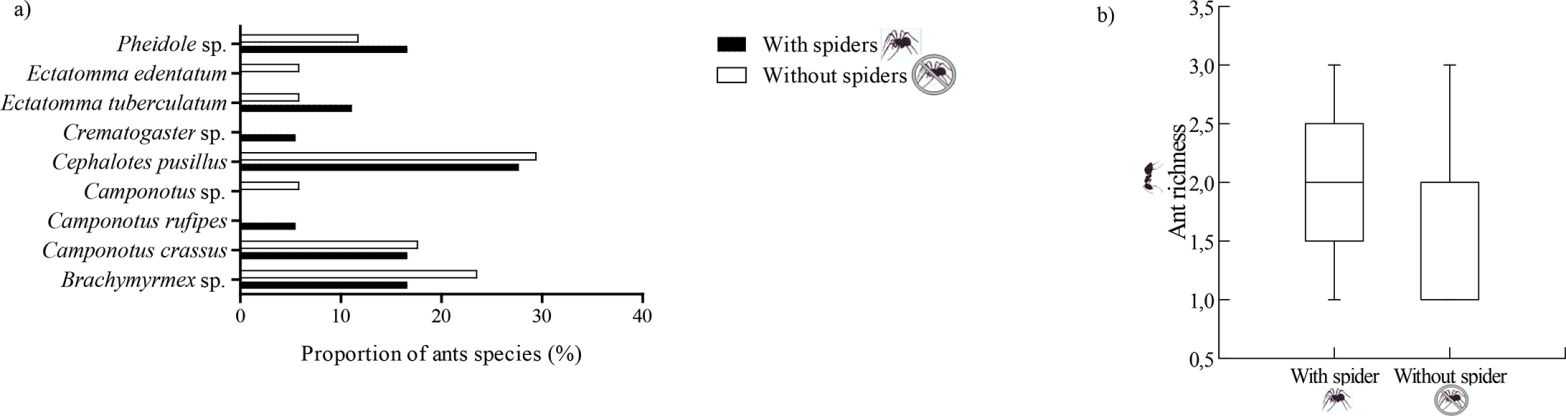
Ant richness. (a) Proportion of different ant species in plants with (control group) and without spiders (ant group), b) Ants richness in plants with (control group) and without spiders (ant group); richness refers to the number of species per tree.

Were observed nine species of ants patrolling the host plant *E*. *gracilipes* (Fig 2*A*). The ant richness did not vary significantly between plants with spiders and plants without spiders (*t* = -0.198, df = 28, p = 0.84; Fig 2*B*). Some species, however, were found in higher proportion in plants without spiders (*Ectatomma edentatum*, *Cephalotes pusillus*, *Camponotus* sp., *Camponotus crassus* and *Brachymyrmex* sp.) (Fig 2*A*).

**Fig 3:**
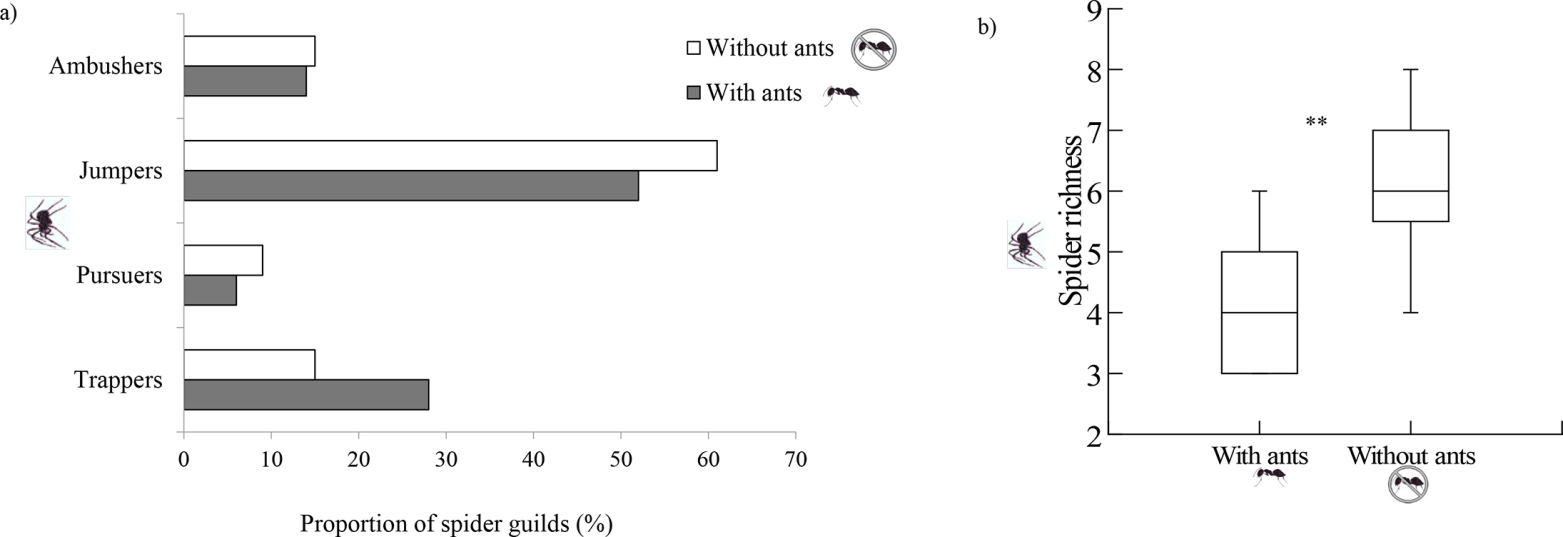
Spider richness. (a) Spiders richness and the proportion of different spider guilds in plants with (control group) and without ants (spider group); (b) spider richness in plants with (control group) and without ants (spider group), richness refers to the number of species per tree. ** indicate significant different—Student's t-test.

A total of 17 morphospecies (from seven different families) of spiders was found in the *E*. *gracilipes* plants. The guild with the highest number of species represented was jumpers (Salticidae N = 6; Oxyopidae, N = 2), followed by trappers (Araneidae, N = 3; Theridiidae, N = 2), ambushers (Ctenidae, N = 1; Thomisidae, N = 2), and pursuers (Anyphaenidae, N = 1). The proportions of spiders belonging to the guilds jumpers, pursuers and ambushers were higher in plants without ants, whereas only the guild trappers showed a higher proportion in plants with ants (Fig 3*A*). The spider richness was significantly higher in plants without ants (*t* = -5.06, *P* = 0.001, df = 28; Fig 3*B*).

**Fig 4:**
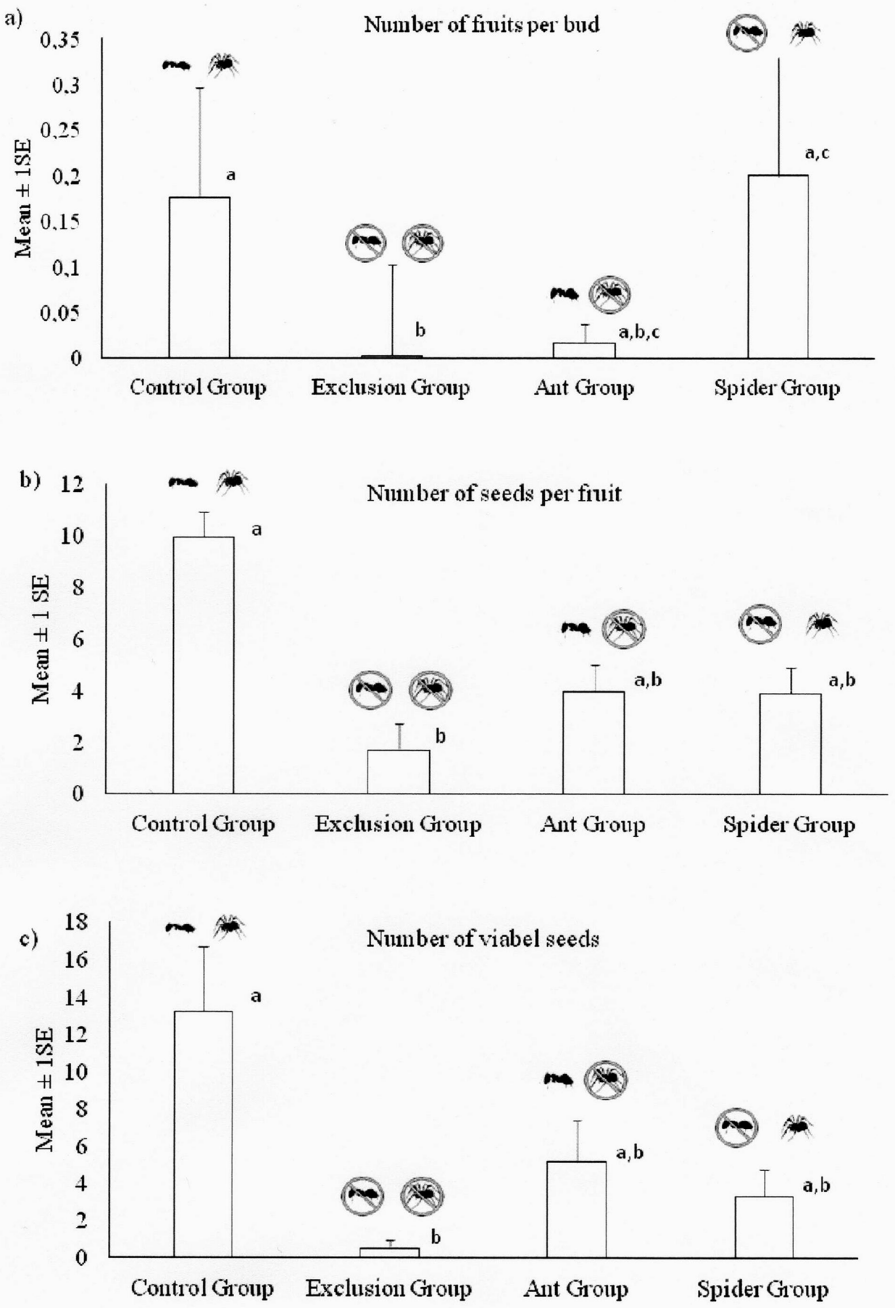
Outcomes of predators on plants. Number of fruits per buds produced (a), number of seed per fruits produced (b) and number of viable seeds (c) in the group ‘Control’—with both predators, ‘Exclusion’–without predators, ‘Ant’–with spiders removed, and ‘Spiders’–with ants removed. Means + 1SE are presented. Different letters over bars point to statistical significant difference (Kruskal-Wallis test, with post-hoc Dunn test; see Table 1).

The fruit set formed per floral buds produced was different among groups (H = 11.17; p = 0.011). Trees occupied only by spiders presented higher fruit production, followed by control, ant and exclusion groups, respectively (Fig 4*A*; Table 1). The production of seeds per fruits was also different among groups (H = 7.41; p = 0.059). However, the paired analysis showed a significant difference only between control and exclusion groups (Fig 4*B*; Table 1). Also the number of viable seeds was different among groups (H = 8.96; p = 0.03, Fig 4*C*), and again the most significant difference was between exclusion and control groups (Fig 4*C*; Table 1).

**Table 1:** Results of Dunn test on data used to compare the effects of spiders, ants and both predators on fruits per bud, seeds per fruits and number of viable seeds on *Eriotheca gracilipes*, see Fig 4.

		Control group	Exclusion group	Ant group
Fruits per bud	Exclusion group	**18.91; p<0.001**	_	_
	Ant group	13.76; p>0.081	-5.154; p>0.163	_
	Spider group	-4.755; p>0.458	**19,15; p<0.011**	9; p>0.118
Seeds per fruit	Exclusion group	**17.02; p<0.006**	_	_
	Ant group	6.519; p>0.162	-7.5; p>0.115	_
	Spider group	-9,019; p>0.07	5; p>0.139	-2.5; p>0.792
Viable seeds	Exclusion group	**15.46; p<0.010**	_	_
	Ant group	7.92; p>0.279	-7.53; p>0.148	_
	Spider group	-9.38; p>0.099	7; p>0.251	-1.46; p>0.639

We found a higher abundance of herbivores in the exclusion and spider groups for all the reproductive phases of *E*. *gracilipes*. In the exclusion group, the most abundant families of herbivores in the flower buds were Proscopiidae, Acrididae and Gryllidae; whereas Gryllidae and Chrysomelidae were most abundant in the flowers. Curculionidae were the most abundant in the fruits. The ant and control groups showed a lower abundance of herbivores. In the fruiting phase, the lowest abundance of herbivores was in control group (Table 2). The total number of herbivores was significant different among groups (H = 14.11; p = 0.0028). However, the paired analysis (Table 2) showed significant differences only between the groups: control and exclusion (Q = 21; p<0.05); ants and exclusion (Q = 18.57;p<0.05); and spiders and exclusion (Q = 8.96;p<0.05).

**Table 2:** Herbivores observed foraging in the buds, flowers and fruits of the *Eriotheca gracilipes* trees visited by ant, spiders, both predators or none. Different letters over total numbers point to statistical significant difference between columns (Dunn test; p < 0.05).

		Control Group		Exclusion Group		Ant Group		Spider Group	
	Family	n	%	n	%	n	%	n	%
Buds									
	Acrididae	3	17.7	7	18.9	4	23.6	3	10.8
	Chrysomelidae	4	23.5	5	13.6	2	11.7	3	10.8
	Lepidoptera larvae	-	-	2	5.4	-	-	4	14.2
	Gryllidae	3	17.7	6	16.2	5	29.5	5	17.8
	Proscopiidae	-	-	8	21.6	-	-	3	10.8
	Curculionidae	3	17.7	4	10.8	3	17.7	1	3.6
	Coreidae	2	11.7	3	8.1	2	11.7	4	14.2
	Pentatomidae	2	11.7	2	5.4	1	5.8	5	17.8
Flowers									
	Acrididae	2	28.6	4	18.2	1	16.7	2	13.3
	Chrysomelidae	3	42.8	6	27.3	2	33.3	5	33.4
	Lepidoptera larvae	-	-	2	9.1	2	13.3	-	-
	Gryllidae	1	14.3	8	36.3	2	33.3	4	26.7
	Pentatomidae	1	14.3	2	9.1	1	16.7	2	13.3
Fruits									
	Coreidae	-	-	2	22.2	4	66.7	-	-
	Curculionidae	2	100	7	77.7	2	33.3	3	100
	Total	26 ^a^		68 ^b^		29 ^ac^		46^ac^	

n = total abundance during study; % = proportion

## Discussion

The hypothesis that spiders and ants exert a positive and complementary effect on the reproduction of *E*. *gracilipes* was confirmed. The beneficial and complementary effects occurred mainly when both spiders and ants were present, leading to an increase in the number of fruits per buds, seeds per fruits and, particularly, in the vitality of the seeds. Therefore, the complementarity of the ecological services provided by different predators, which possess different abilities and cognitive and predatory capacities, may result in a direct benefit to the adaptive value of the host plant. A similar pattern has been reported for wasps that visit extrafloral nectaries (e.g. [8, 43, 44]). The importance of these complementary effects was also confirmed by the significant reduction in the number of herbivores in plants visited by both, ants and spiders. However, the results of these interactions may still be ambiguous, as shown by Nahas and co-workers [11], who did not find a significant positive impact when ants and spiders acted together or separately in the reproduction of *Q*. *multiflora* (Vochysiaceae); rather, showed a complement impact of spiders and ants on herbivory of the vegetative structures of the plant. Conversely, Ruhren and Handel [45] reported that, in the *Chamaecrista nictitans* (Fabaceae) shrub, spiders of the family Salticidae chose plants with active extrafloral nectaries to house them. These authors also observed these spiders feeding on nectar. In our study more fruits where formed in plants visited by ants and spiders simultaneously. However, plants visited only by spiders also produced more fruits than plants visited only by ants. Assunção and co-workers [46] showed that in ants may repel pollinators and reduce fruit formation in plants of Brazilian savanna.

The role of generalist predators, such as spiders and ants, using the same foraging substrate, may result in competition for prey, and these predators may attack one another. We observed that the spider richness in the absence of ants was significantly higher, whereas the ant richness in the absence of spiders did not vary significantly. This difference in our results may be attributed to the composition of distinct species of both ants and spiders and, consequently, to the different types of interactions among them. Nahas and co-workers [11] found that the ant abundance and richness were similar in the presence and absence of spiders; however, the spider abundance and richness were lower, although not significantly, in plants visited by ants. Sanders and co-workers [47] reported a 90% decrease in the density of ants in the presence of web-building spiders, a 50% decrease in the density of herbivores in the presence of web-building spiders or hunter spiders, and a 60% decrease when both groups of spiders were present. Hunter spiders (jumpers, ambushers and pursuers) were found in higher proportions in plants without ants. This result must be associated with the behavior of these spiders, which move constantly through the vegetation, using it directly for foraging and also for shelter and reproduction. Such movement increases their encounters with ants. Opposite, web-building spiders (trappers), which were found in a higher proportion in plants with ants, are usually inaccessible to ants.

Predators affect herbivores directly, due predation, or indirectly, reducing access to food sources for example [11]. Distinct predators represent different hunting strategies, and in the system studied the diversity of both ants and spiders in *E*. *gracilipes* trees was great. Thus, their action simultaneously and separately, can be one of the main explanations for the reduction of the number of herbivores, which resulted in high values for the viability of the *E*. *gracilipes* seeds. Rosa [48] observed that the leaf area consumed on *Hirtella myrmecophila* (Chrysobalanaceae) was consistently larger in the leaves on which *Dipoena bryantae* (Araneae) spiders were present. This suggests that the presence of the spider negatively affects the behavior of the ant *Allomerus octoarticulatus* (Myrmicinae), reducing its efficiency in defending the plant. Another study of the Amazonian myrmecophyte species *Maieta poeppigii* (Melastomataceae) demonstrated that after an herbivory event, the number of ants present in the leaves increases in response to the presence of herbivores and volatile compounds that are released from the damaged leaf [49]. Similarly, spiders can sense volatile compounds and are attracted to the leaves with a higher concentration of ants. In fact, some studies have demonstrated that the release of volatile compounds induced by the consumption of leaves attracts predators of herbivores [50, 51]. According to Nelson and Jackson [52], juveniles of the spider *Evarcha culicivora* (Salticidae), which feed on nectar, are attracted by the release of phytochemical compounds of the host plant *Lantana camara* (Verbenaceae), such as β-caryophyllene and α-humulene, highlighting strategies promoted by host plants to attract potential predators of its herbivores. In the adult phase, *E*. *culicivora* does not feed on nectar but rather on insects, also using the plant as a mating site.

## Conclusions

The result of the joint action of spiders and ants, however, varies according to their synergistic and antagonistic frequencies [53]. In our study, the proportion of the reduction in the number of herbivores was more significant when ants and spiders were present together, reflecting positively on the plant’s reproduction. Therefore, spiders and ants are active at the third trophic level and may indirectly affect the reproductive value (number of seeds and their viability) and, consequently, the fitness of the plants they visit.
